# Discontinuous Translocation of a Luciferase Protein beyond Graft Junction in Tobacco

**DOI:** 10.14252/foodsafetyfscj.D-23-00010

**Published:** 2024-03-22

**Authors:** Taira Miyahara, Hitomi Ohkubo, Yukiko Umeyama, Taichi Oguchi, Takumi Ogawa, Daisaku Ohta, Tomofumi Mochizuki, Hiroaki Kodama

**Affiliations:** 1 Graduate School of Horticulture, Chiba University, 1-33 Yayoi-cho, Inage-ku, Chiba 263-8522, Japan; 2 Institute of Life and Environmental Sciences, University of Tsukuba, 1-1-1 Tennodai, Tsukuba, Ibaraki 305-8572, Japan; 3 Tsukuba Plant Innovation Research Center, University of Tsukuba, 1-1-1 Tennodai, Tsukuba, Ibaraki 305-8572, Japan; 4 Graduate School of Agriculture, Osaka Metropolitan University, 1-1 Gakuen-cho, Naka-ku, Sakai,Osaka 599-8531, Japan; 5 Graduate School of Life and Environmental Sciences, Osaka Prefecture University, 1-1 Gakuen-cho, Naka-ku, Sakai, Osaka 599-8531, Japan

**Keywords:** luciferase, new plant breeding technology, protein translocation, tobacco, transgrafting

## Abstract

Transgrafting, a grafting technique that uses both genetically modified (GM) and non-GM plants, is a novel plant breeding technology that can be used to improve the efficiency of crop cultivation without introducing foreign genes into the edible parts of non-GM plants. This technique can facilitate the acquisition of disease resistance and/or increased yield. However, the translocation of low-molecular-weight compounds, ribonucleic acid (RNA), and proteins through graft junctions raises a potential safety risk for food crops. Here, we used a transgenic tobacco plant expressing a firefly luciferase gene (*LUC*) to examine the translocation of the LUC protein beyond the graft junction in grafted plants. We observed the bi-directional translocation of LUC proteins in transgrafted tobacco plants, i.e., from the rootstock to scion and vice versa. Transcriptomic analysis revealed that transcripts of the LUC gene were undetectable in non-GM plant bodies, indicating that the LUC protein itself was translocated. Moreover, the movement of the LUC protein is an episodic (i.e., non-continuous) event, since non-GM samples showing high LUC activity were flanked by non-GM samples showing no apparent LUC activity. Translocation from the GM to non-GM part depends on the characteristics of GM plant bodies; here, the enhanced translocation of the LUC protein into the non-GM scion was observed when LUC-expressing rootstocks with hairy roots were used. Moreover, the quantity of translocated LUC protein was far below the level that is generally required to induce an allergenic response. Finally, since the LUC protein levels of plants used for transgrafting are moderate and the LUC protein itself is relatively unstable, further investigation is necessary regarding whether the newly expressed protein in GM plants is highly stable, easily translocated, and/or highly expressed.

## 1. Introduction

Grafting is a traditional technique in which two different plant bodies are merged into one plantlet via bonding along the cutting surface. This technique has been used for thousands of years for the cultivation of fruit crops^1^^)^. Wild plants or cultivars that are resistant to biotic and abiotic stresses are often used as rootstocks, and cultivated varieties that possess good food properties—but which may be sensitive to abiotic and/or biotic stress—are used as scions. In the 1850s, a soil-dwelling insect pathogen originating in the United States, phylloxera, invaded European grape plants, thereby causing serious damage to the grape and wine industries. This pest was overcome via grafting of phylloxera-sensitive European grape cultivars onto phylloxera-resistant American grape rootstocks^2^^)^. Moreover, grafting has also been employed to reduce farm labor requirements. For example, the dwarf apple rootstock has been used to improve fruit harvest efficiency, namely by altering tree morphology to modify shoot elongation and blanch angle^3^^)^. In this case, phenotypic alteration to the scion is likely established by the exchange of biomolecules—including small-molecule chemicals, RNAs, and proteins—between the rootstock and scion via the graft junction^4^^)^.

Transgrafting is a technique that produces a plant consisting of a genetically modified (GM) rootstock and a non-GM scion, or vice versa^5^^)^, and is a key new plant breeding technology (NPBT)^6^^,^^7^^)^. GM rootstocks with increased resistance to abiotic and/or biotic stresses are designed to attenuate decreases in crop production under suboptimal conditions. Moreover, since fruits and crops obtained from the non-GM plant parts of the transgrafted plant do not contain transgene sequences in their genome, the consumption of these foods is expected to be exempted from regulations for GM plants. However, as mentioned above, the possible exchange of biomolecules between the rootstock and scion raise concerns regarding the safe use of foods obtained from grafted plants. For example, one incident occurred in which eggplant (*Solanum melongena*) fruits were harvested from plants grafted onto *Datura metel* rootstock; when served they caused severe food poisoning in Japan^8^^)^. This incident was caused by the transport of toxic alkaloids from the *Datura* rootstock to the eggplant fruits on the scion. Alkaloids, frequently produced by members of the Solanaceae family, are known to be synthesized in the roots then transported throughout the plant^9^^)^. Therefore, if toxic compounds are produced in a transgene-dependent manner, the fruits and crops obtained from the non-GM scions of transgrafted plants may cause health risks if consumed.

In addition to the translocation of low-molecular compounds via the graft junction, protein translocation has been also observed. Two types of protein movement have been studied: short-distance movement between neighboring cells via plasmodesmata and long-distance movement in which proteins are translocated to distal tissues via the vascular system. Plasmodesmata are tubes with diameters ranging from 30–60 nm; this tube size is often regulated by the deposition of β-1,3-glucan polymers (i.e., callose) to cell walls containing plasmodesmata. Plant cells control the permeability of biomolecules through plasmodesmata to regulate their cell-to-cell transport^10^^)^. Moreover, it has been observed that plasmodesmata can form across a graft junction^11^^)^. Green fluorescent protein (GFP) is used as a soluble reporter, and has been found to be capable of diffusive spreading into neighboring cells via plasmodesmata^12^^)^. Long-distance protein movement was first demonstrated in *Arabidopsis*, in which the floral signaling protein FLOWERING LOCUS T (FT) was found to be transported over long distances via the phloem^13^^)^. Subsequently, FT homologous proteins were found and their translocation tracked in various plant species, including potato^14^^)^, tomato^15^^)^, and squash^16^^)^. Moreover, grafting was frequently used to demonstrate the long-distance translocation of FT proteins. In addition, long-distance movement of GFP-tagged chloroplast transit peptides from the scion to the rootstock has also been detected in transgrafted *Arabidopsis* plants^17^^)^.

In previous studies we performed omics analyses of the edible parts of homo-transgrafted plants^18^^,^^19^^)^. Homo-transgrafting refers to a grafting technique in which a non-GM scion and GM rootstock, or vice versa, are prepared from the same plant species. The GM tomato^18^^)^ and GM potato^19^^)^ plants used in previous transgrafting experiments harbored transgenes encoding β-glucuronidase (GUS) and a potato FT homolog protein, respectively. In the resulting tomato fruits and potato tubers, the newly expressed GUS and FT proteins were not detected by proteomic analyses. Next, we subjected tomato fruits harvested from hetero-transgrafted plants to omics analysis^20^^)^. In this case, hetero-transgrafting was established using a GM tobacco rootstock and a non-GM tomato scion. Here, a proteomic analysis of tomato fruits detected two kinds of tobacco proteins. This result and a previous report by Paultre *et al.* (2016) raised the possibility of the long-distance movement of newly expressed proteins (NEPs) produced in the GM parts of transgrafted plants. Since the allergenicity of NEPs is the most important assessment issue for the guidelines for GM foods prepared by the Codex Alimentarius Commission (CAC)^21^^)^, the understanding of the long-distance movement of NEPs from GM plant parts—and especially the levels of translocated NEPs and their distribution patterns in non-GM parts—is critically important to establish the safety of transgrafted plants. In this study, we used a GM tobacco plant that expresses firefly luciferase (LUC) gene for producing a homo-transgrafted tobacco plant. Since LUC activity can be detected with quite high sensitivity, even very low levels of the LUC protein are easily detected. In addition, the short turnover of LUC proteins makes them ideal for monitoring translocation, where the LUC protein produced in the GM plant body accumulates at specific parts in the non-GM plant body. The results obtained here show that the LUC protein moved from the rootstock to the scion, and vice versa. Next, we investigated the effects of so-called “mentor-grafting^22^^)^” on LUC protein translocation. Mentor grafting is a technique in which the growth of scion parts is preferentially dependent on the supply of nutrients from the rootstock by the removal of all scion leaves except young leaves near the scion’s shoot apical meristem. The transport of biomolecules from rootstock to scion is expected to be enhanced under mentor-grafting conditions relative to conventional grafting. For example, the enhanced movement of small RNAs has been observed in mentor-grafted tomato plants^23^^)^. Furthermore, we also addressed the translocation of LUC protein when the roots of the rootstock were replaced with hairy roots. Hairy roots are the transformed organ formed following infection by *Agrobacterium rhizogenes* (*Rhizobium rhizogenes*). A modified morphological phenotype has been reported in cherry scions grafted onto a rootstock that had been regenerated from hairy roots^24^^)^. Therefore, the effects of the rootstock on the phenotype of the scion may be greater when using a rootstock with hairy roots than when using a rootstock with normal roots. Here, this transgrafting study used a GM tobacco plant expressing the *LUC* gene to reveal that the translocation of the LUC protein beyond the graft junction is dependent on the grafted condition and is also markedly affected by rootstock characteristics. The intermittent localization of LUC proteins in the scion indicated that the potential risks of the transgene product should be assessed even if the transgene products are not detected in the samples obtained from the non-GM plant bodies of the transgrafted plants.

## 2. Materials and Methods

### 2.1 Plant Materials and Grafting

A transgenic tobacco line expressing the *LUC* gene (Uniprot Accession: P08659) was produced as previously described^25^^)^. This transgenic plant was named the “LUC plant.” Transgrafted plants were prepared using LUC plants and corresponding non-GM, wild-type (WT) tobacco plants (i.e., *Nicotiana tabacum* cv. SR1). Transgrafting was performed using a conventional grafting method. Briefly, seeds of WT and LUC plants were sown under sterile conditions and one month later the resulting seedlings were transferred to soil. Rootstocks were prepared by cutting the stem at a position 10−20 cm above the soil, and shoots with three mature leaves were used as scions. The grafted junctions were then fastened with surgical tape. Combinations of grafted WT and LUC plants are described using the format “scion/rootstock.”

**Fig. 1.**
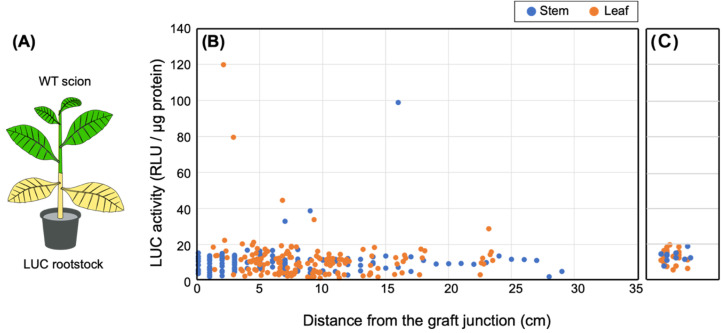
Movement of the luciferase (LUC) protein from the rootstock to scion. (A) Transgrafting diagram. Transgenic LUC plants were used as the rootstock (shown in yellow) and WT plants were used as the scion (green). (B) LUC activity in scions. Activity was measured at 3, 4, 6, 8 WAG. All data are combined. The numbers of measurements used for stem and leaf samples were 137 and 132, respectively. (C) Background fluorescence levels during the LUC assay. LUC activities were determined using samples prepared from stems and leaves of WT plants. The numbers of measurements used for stem and leaf samples were 10 and 20, respectively.

In WT (scion)/LUC (rootstock) grafted plants (Fig. 1), axillary buds that formed on the rootstock were removed so that nutrients from the rootstock were efficiently transported to the scion. We prepared a total of 13 transgrafted plants. Then, approximately 3–8 weeks after grafting (WAG), we examined the LUC activities of stem samples collected at 10 mm intervals from the graft junction. As a control, LUC activity was also measured in the leaves and stems of 2 month-old WT plants. We also harvested and evaluated the LUC activity of all scion leaves.

**Fig. 2.**
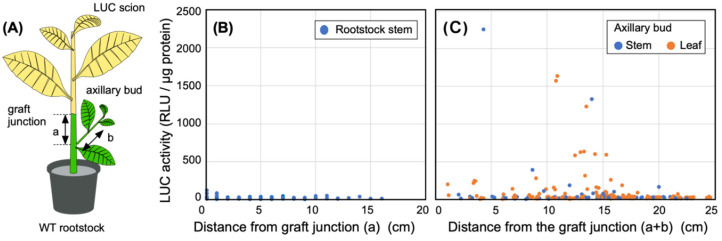
Movement of the LUC protein from the scion to rootstock. (A) Transgrafting diagram. Transgenic LUC plants were used as the scion (shown in yellow) and WT plants were used as the rootstock (green). The distance from the graft junction was determined as illustrated. (B) LUC activity in the stem of the WT rootstock. The number of measurements used for the stem samples was 91. (C) LUC activity in newly emerged axillary buds on WT rootstocks. The number of measurements used for stem and leaf samples were 56 and 147, respectively. LUC activity was measured at 3 WAG. All data are combined and shown in one figure.

We prepared a total of eight transgrafted plants (i.e., LUC (scion)/WT (rootstock) plants; Fig. 2). At 3 WAG, non-GM stem samples were collected along a transect at each 10 mm interval from the graft junction. Moreover, we also collected samples from the stems of axillary buds formed on the rootstock at intervals of 10 mm. All leaves from the axillary buds were sampled to measure LUC activity.

### 2.2 Transgrafting Using the Mentor Grafting Method

**Fig. 3.**
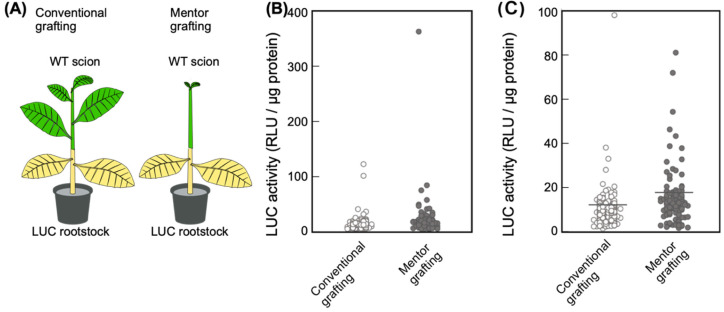
Movement of the LUC protein from the scion to rootstock in mentor grafting. (A) Diagram of mentor grafting. LUC plants were used as the rootstock (shown in yellow) and WT plants were used as the scion (green). In the mentor grafting, most of the developed leaves of the scion were removed after the scion was grafted onto the LUC rootstock. (B) LUC activity of the scion. LUC activity was measured at 3 WAG. (C) LUC activity measurements under 100 RLU/μg protein are shown. The number of measurements used for conventional and mentor grafting were 195 and 144, respectively. Mean LUC activity levels were 11.5 and 18.3 for the conventional and mentor graftings, respectively. Means are shown using a bar.

Mentor grafting is a method in which all leaves on the scion except the youngest, near the shoot apical meristem, are removed^22^^)^. A total of 10 scions were grafted onto LUC rootstocks, and for five of these plants all developed leaves were then removed (i.e., mentor grafting). The remaining five grafted plants were grown as negative controls (i.e., conventional grafting) (**Fig. 3**). Stem and leaf samples from all plants were collected at 3 WAG. Any axillary buds formed on the scion during this period were removed.

### 2.3 Preparation of WT/LUC Transgrafted Plants with Hairy Roots

**Fig. 4.**
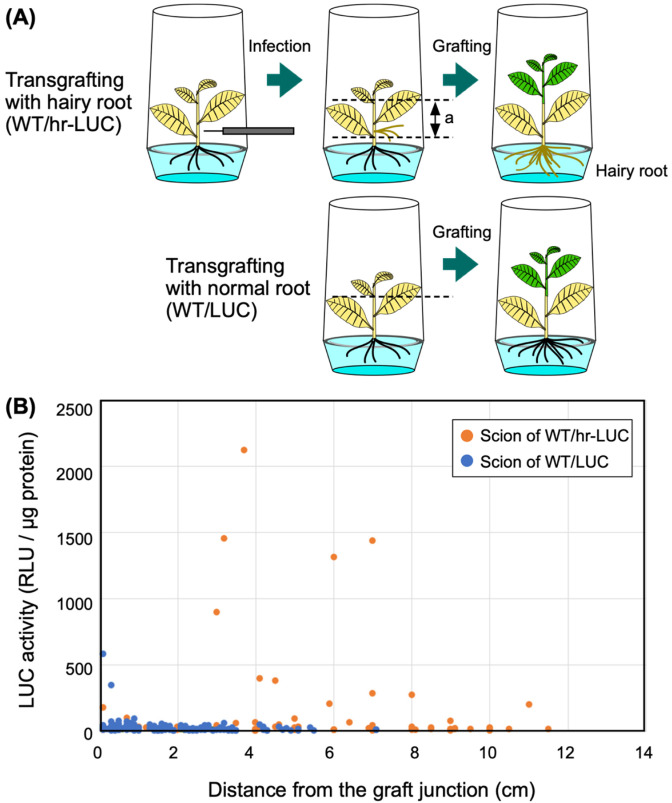
Effects of hairy root rootstocks on the movement of LUC protein to the scion in transgrafted tobacco. (A) Diagram of the preparation of transgrafted plants with *LUC-*expressing hairy roots. Transgenic *LUC* plants (shown in yellow) were infected with *Agrobacterium tumefaciens* R-1000 strain. This strain has an Ri plasmid instead of a Ti plasmid, and induces hairy roots at the infected site. After the emergence of hairy roots, plant bodies with hairy roots (shown as “a” in the figure) were transferred to a new sterilized medium, then used as a rootstock. The resulting transgrafted plants with hairy roots were designated as WT/hr-LUC. In a control experiment, transgrafted plants (WT/LUC) were prepared under sterile conditions. (B) LUC activity of scions. Grafted plants were transferred to soil at 3 WAG and were further cultured for 3–8 weeks. Next, the LUC activity of the scion (including both stem and leaf samples) was assessed. The numbers of measurements for the WT/hr-LUC and WT/LUC transgrafted plants were 76 and 163, respectively.

Hairy roots were generated via infection of *Agrobacterium tumefaciens* R-1000 strain. This strain carries a hairy-root-inducing Ri plasmid, pRiA4b, instead of Ti plasmids^26^^,^^27^^)^. For this experiment, two-month-old LUC plants grown in plant culture vessels were infected by pricking using a syringe needle containing *Agrobacterium* cells. The prick occurred on the stem 2−3 cm above the root, and hairy roots usually formed two weeks after infection. Next, normal roots were removed by cutting the stems of LUC plants just below the site of hairy root formation. The resulting LUC plants with hairy roots (called “hr-LUC plants”) were transferred to a Murashige-Skoog solid medium containing 750 mg/L Augmentin (GlaxoSmithKline, Brentford, UK) then grown in plant culture vessels. After 3 weeks, aseptically grown WT scions were grafted onto hr-LUC rootstocks (**Fig. 4**). Similarly, LUC plants grown in plant culture vessels were used for grafting onto WT plants. The numbers of the WT/hr-LUC and WT/LUC transgrafted plants were 5 and 13, respectively. The leaves of two WT/hr-LUC plants were harvested at 3 WAG. The other three WT/hr-LUC plants were transferred on soil on the same day, then subsequently cultured for another three weeks. We obtained leaf and stem samples on 4, 6, and 8 WAG, and subjected these samples to measurements of LUC activity.

### 2.4 Determination of LUC Activity

All sampled tissues were immediately measured for LUC activity. We used the Luciferase Assay System (Promega, WI, USA) to quantify LUC activity. Briefly, 200 µL of fivefold diluted Cell Culture Lysis Reagent was added to each sample. After homogenization, all samples were centrifuged at 15,000 rpm for 10 min at 4°C. The LUC activity of the supernatant was determined as previously described^28^^)^. The assay was performed in 1 sec raw format using a Lumat3 LB7608 (Berthold Technologies GmbH & Co. KG, Bad Wildbad, Germany). All results are shown as relative light units (RLUs). The calibration curve for luciferase protein quantification was prepared by using a purified luciferase protein standard (Merck Millipore, Billerica, MA, USA, Code number: L9420-1MG).

### 2.5 Protein Quantification

Protein quantification was performed using a DC protein assay (Bio-Rad Laboratories, Inc. CA, USA). Calibration curves were prepared via stepwise dilution of bovine serum albumin in sterile water. The assay system involved a small-scale modification of the micro assay protocol; briefly, 20 µL of reagent S was added to 1 mL of reagent A to create reagent A’. Next, 70 µL of reagent A’ was added to 140 µL of diluted sample and vortexed. Subsequently, 560 µL of reagent B was added and vortexed well before being incubated at room temperature for 15 min. The absorbance at 750 nm was then measured with a spectrophotometer (UV-1800, SHIMADZU, Kyoto, Japan).

### 2.6 Transcriptomic Analysis of the Transgrafted Scion

To perform transcriptomic analyses, total RNA was first extracted from frozen leaf and petiole samples of WT/LUC plants prepared by a conventional grafting method. Leaves and petioles were frozen in liquid nitrogen immediately after sampling. They were then used for RNA extraction. Extraction was carried out using a FavorPrep Plant Total RNA Mini Kit (Favorgen Biotech Corp., Taiwan). The outsourcing service of Eurofins Genomics (Tokyo, Japan) constructed the RNA library and obtained mRNA sequencing data. Briefly, the mRNA was purified as poly(A)^+^ RNA, and paired-end 150-base sequencing data was generated using a NovaSeq 6000 platform (Illumina Inc., San Diego, CA, USA). The mRNA-seq dataset (BioProject ID: PRJDB11010, Experiment ID: DRX411439-40) contained a total of 161.7 million reads. Adapter sequences were then trimmed, and low-quality reads containing poly-N sequences and/or that were shorter than 50 bp in length were discarded using fastp version 0.23.4. The bowtie2 version 2.5.1 alignment tool was then used to search for *LUC* gene transcripts and align reads to *Nicotiana tabacum* cDNA (Ntab-TN90_AYMY-SS_NGS.mrna.annot.fasta).

## 3. Results

### 3.1 Detection of LUC Activities in the Scion of WT/LUC Plants

A homozygous transgenic tobacco line expressing the firefly *LUC* gene was used for transgrafting experiments. When the LUC plants were used as the rootstock and WT plants as the scion, the resulting grafted plants were called WT/LUC plants. Plants grown on the soil were used for grafting. Moreover, we harvested scion samples 3, 4, 6 and 8 WAG, and all LUC activities of the scion were determined collectively (**Fig. 1**). Interestingly, we detected weak LUC activity in the (WT) scion, and this activity was distinguishable from the background luminescence of the WT plants (i.e., approximately 0−20 RLU/μg protein). The highest LUC activity in the scion was observed 4 WAG; thereafter the LUC activity was detected only at a relatively low level (**Fig. S1**). The highest LUC activity (119 RLU/μg protein) was observed in leaves that had been harvested 3 cm above the graft junction. Furthermore, despite the fact that the scion stem samples (1 cm in length) were prepared in series from the graft junction, the strength of LUC activity did not fade in a linear manner but rather had seemingly random peaks. In fact, the second highest LUC activity (99 RLU/μg protein) was observed in a stem sample prepared 16 cm above the graft junction. In addition, we did not detect increased LUC activity in seeds produced by the scion of WT/LUC plants (**Fig. S2**).

Transcriptomic analysis was then carried out on the scion leaves and petioles of WT/LUC plants at 6 WAG. Transcriptomic data for two samples were generated, and we obtained approximately 80 M reads for each. Next, alignment of these reads to the cDNA database generated using *Nicotiana tabacum* genome data resulted in alignment rates of around 90% for each sample (Table S1; WT-LUC-1 read data was obtained from petiole samples and WT-LUC-2 was obtained from a mix of petiole and leaf samples). The remaining 10% of reads could not be aligned to any tobacco cDNAs and these sequence reads may correspond to RNA species such as tRNA, rRNA and so on or RNAs isolated from environmental contaminants such as bacteria and fungi in the samples. The sequencing of the *Nicotiana tabacum* genome is not yet complete. The number of publicly available tobacco genes is very large because the tobacco database contains redundant sequences. A total of 189,413 genes (as cDNA references) can be found in the tobacco cDNA database, and the transcriptomic reads from the scion samples were mapped to the 137,645 genes, indicating that the transcriptomic reads covered approximately 73% of the tobacco cDNAs. Since the scion samples consisted of petiole and leaf tissues, the genes preferentially expressed in the reproductive organs and roots would not be detected in the transcriptomic reads of the scion samples. Under this condition, no transcriptomic reads were aligned to the *LUC* genes. However, the possibility remains that *LUC* gene transcripts are translocated from the rootstock to the WT scions of WT/LUC plants.

### 3.2 LUC Activity in the Rootstocks of LUC/WT Plants

Next, we determined which direction—i.e., from rootstock to scion or vice versa—was more common for LUC protein translocation. For this purpose, a LUC scion was grafted onto a WT rootstock, and this grafted plant was called a “LUC/WT plant.” Next, LUC activity was measured in the stem part of the rootstock and in new axillary buds that formed on the rootstock after grafting (**Fig. 2**). Interestingly, a number of stem and leaf samples obtained from the axillary buds on the rootstock showed very high LUC activities (i.e., more than 500 RLU/μg protein) (**Fig. 2C**). In fact, the highest LUC activity (2,200 RLU/μg protein) observed in the axillary buds was 18-fold higher than the highest activity observed in the scion of the WT/LUC transgrafted plants (**Fig. 1**). In contrast, stem samples taken from directly below the graft junction showed almost no LUC activity and resembled the background luminescence level (**Fig. 2B**). Therefore, the LUC protein produced in the scion was observed to move through the stem of the rootstock and accumulated in young tissues such as axillary buds.

We then assessed whether the LUC protein moved more frequently from scion to rootstock than vice versa. Although the *LUC* gene is transcribed under the control of the Cauliflower mosaic virus 35S promoter and is therefore constitutively expressed throughout all plant tissues, transcription itself is more active in young than in old tissues. Therefore, actively growing, young tissues are expected to show higher LUC activity than older tissues. When LUC activity was quantified in the LUC scion and LUC rootstock that were used for transgrafting, the scion showed increases in LUC activity of approximately 1.4-fold and 2.6-fold for stem and leaf samples, respectively, relative to corresponding samples from the rootstock (Fig. S3). In contrast, the LUC activities detected in the axillary buds of the rootstocks of LUC/WT plants were significantly higher (maximum 2,200 RLU/μg protein) than those detected in the scion samples of the WT/LUC plants (maximum 119 RLU/μg protein). Therefore, even though LUC protein production was expected to be higher in the scion than the rootstock, it is likely that the LUC protein moves more easily from the scion to the rootstock than vice versa.

### 3.3 Effects of the Mentor Grafting Technique on the Movement of LUC Protein

When using the mentor grafting technique, scion growth largely depends on the nutrient supply from the rootstock. Next, we determined whether movement of the LUC protein is enhanced in mentor-grafted WT/LUC plants. Compared to the LUC activity of WT/LUC plants prepared using the conventional grafting, we detected slightly higher LUC activities in the scions of WT/LUC plants produced by mentor grafting (**Fig. 3**). Specifically, we observed a mean value of 10.2 RLU/μg protein for conventional grafting and 18.3 RLU/μg protein for mentor grafting. Furthermore, in the mentor grafting plants we detected a significantly high LUC activity (360 RLU/μg protein) in stem samples taken 3 cm above the graft junction. Taken together, these results indicate that the movement of the LUC protein is influenced by the grafting method, but its effect on the quantity of LUC protein that is moved remains quite limited.

### 3.4 LUC Activities of WT Scions Grafted onto LUC Rootstocks with Hairy Roots

Hairy roots are root organs transformed with the Ri plasmid. The characteristics of hairy roots include rapid growth and a high capacity for secondary metabolite and recombinant protein production^29^^,^^30^^)^. Next, we studied whether or not the movement of LUC protein from the rootstock to the scion was strengthened by the hairy root phenotype. To do so, LUC plants were first infected with *Agrobacterium* harboring an Ri plasmid. After generating hairy roots at the infection site, normal roots were removed and the resulting LUC plants with hairy roots (called “hr-LUC plants”) were used as a rootstock (**Fig. 4A**). We used LUC plants with normal roots as a control. We detected LUC activity measurements above 200 RLU/μg protein in scion samples of WT/hr-LUC plants taken from more than 3 cm above the graft junction. The highest LUC activity (i.e., 2,118 RLU/μg protein) was detected at a distance of 3.7 cm from the graft junction (**Fig. 4B**). Furthermore, we also detected high (201 RLU/μg) LUC activity in a scion sample taken from 11 cm above the graft junction. We note that in this experiment, grafting was carried out using aseptically grown, young plants (**Fig. 4A**), which may enhance the movement of the LUC protein. Two high LUC activities (i.e., 345 and 583 RLU/μg protein) were detected in control samples prepared from the stem tissues close to the graft junction.

The highest LUC activity observed in the scion of WT/hr-LUC plants (Fig. 4B) was comparable to the highest value observed in the axillary buds that emerged from the rootstock of LUC/WT plants (Fig. 2C). In stem samples prepared from the scions of WT/hr-LUC plants, we found that proximal samples and distal samples flanking other samples with extremely high LUC activity did not consistently show high LUC activity (Fig. S4). These results confirms that LUC protein movement across the graft junction was episodic instead of continuous.

## 4. Discussion

Risk assessments for the safe use of GM foods are mandatory in most countries, including Japan. Moreover, the evaluation of the allergenic and toxic potential of NEPs in GM foods is critically important^31^^)^. Thus, the ability to detect transgene sequences is a prerequisite for adequate risk management of GM foods. Therefore, when particular fruits obtained from the scion parts of transgrafted plants do not contain any transgene sequences in their genome, these foods should be out of scope of risk management strategies. However, if NEPs move beyond the graft junction, we must consider their allergenic and toxic potential before the use of food products obtained from transgrafted plants is sanctioned. Here, we showed that a NEP (i.e., LUC protein) was detected in WT plant bodies of transgrafted plants.

Small molecular compounds, such as alkaloids, can be quantitatively detected, and the effects of transgrafting on their abundance in fruits has also been observed. For example, α-tomatine, a toxic substance found in tomato, was less abundant and nicotine, a toxic substance found in tobacco, was more abundant in tomato fruits grown on transgrafted plants in which a non-GM tomato scion was grafted onto a GM tobacco rootstock^20^^)^. In contrast, our data showed that the LUC protein was episodically detected in the WT plant bodies of transgrafted plants. In fact, the non-GM stem samples that showed extremely high LUC activity were flanked by stem tissues that did not show any significant LUC activity (Fig. S4). Similar results have been reported for the translocation of GFP fusion proteins from transgenic scions to the specific tissues of the non-GM roots^17^^)^. These results suggest that the movement of the LUC protein beyond the graft junction occurred episodically, and not continuously, in transgrafted plants. The firefly LUC protein is relatively unstable and has a half-life of approximately 3−4 h^32^^)^. Due to this relative short half-life, LUC has been used for various circadian studies^33^^)^. If so, we detected the LUC protein relatively shortly after translocation into non-GM plant bodies from the GM parts of transgrafted plants. Although the route of LUC protein movement has not yet been determined, the LUC protein most likely moves via phloem, as does the FT protein. The velocity of FT in the phloem has been estimated to be 30–50 cm h^–1^, which is comparable to the estimated velocity of the phloem flux^34^^,^^35^^)^. Therefore, it is likely that the export of LUC protein from GM plant bodies is an episodic event that occurs frequently. In contrast, the preferential detection of LUC activity in the axillary buds of the rootstock and not in stem tissues that directly abutted the GM scion (Fig. 2) suggest that the LUC protein moved beyond the graft junction to accumulate at specific “sink tissues.” Indeed, Paultre et al. have suggested that GFP fusion proteins translocated through the phloem are “unloaded” from the phloem in specific tissues^17^^)^. In addition, the tissues in which the translocated proteins accumulated differed among the different GFP fusion proteins, suggesting that phloem unloading may be regulated differently for each protein^17^^)^. If such a protein export and unloading mechanism is associated with most of the translocated protein types, the discontinuously detected LUC protein may be explained by phloem unloading at the specific plant body and may not by the episodic export from the GM plant body.

In WT-LUC transgrafted plants, we observed that the mentor grafting technique showed only a limited effect on the transport of LUC protein from the rootstock (Fig. 3). Moreover, high levels of LUC protein were detected in non-GM scions when the hr-LUC rootstock was used for grafting (Fig. 4). These results suggest that the level of NEPs in the non-GM scion was dependent on the characteristics of the rootstock that produced the NEPs. In contrast, the effect of scion condition on NEP translocation was nearly negligible, since mentor grafting has quite a limited effect. Hairy roots are well known to grow vigorously and produce secondary metabolites and NEPs^29^^,^^30^^)^. However, our results also suggest that hairy roots can systemically export NEPs more efficiently than normal roots. In conventional grafting, those rootstocks that show maximum stress-resistance and rooting vigor are preferably chosen, especially for heterografting. Our results indicate that the NEP levels of non-GM scions can be affected by the GM rootstock, and it is therefore necessary to assess what kind of GM rootstock is used for grafting, especially for hetero-transgrafting.

In this study, the average measured LUC activity was approximately 280,000 RLU/μg protein in the young leaves of LUC plants (Fig. S3), and the highest detected LUC activity in non-GM plant bodies was approximately 2,000−2,500 RLU/μg protein (Figs. 2 and 4). When we used purified firefly luciferase as a calibration standard for protein quantification, we obtained a maximum estimate of 0.51 pg total luciferase protein per sample for the non-GM samples (stem or leaf samples) which showed the highest LUC activity of all samples tested. The lowest observed adverse-effect level (LOAEL) has been estimated for many IgE-dependent food allergies, and LOAELs are commonly in the range of 1-2 mg of natural foods, representing a few hundred micrograms of protein^36^^)^. Therefore, the amount of LUC protein that moved though the graft junction is very small when compared with the LOAELs of IgE-dependent food allergens. In addition, although LUC activity was not detected in seeds harvested from the non-GM scions, tobacco rootstock-derived proteins have been detected in tomato fruits in our previous study^20^^)^. As discussed above, it is possible that the tissue in which the translocated proteins accumulate differs among the translocated protein types. Therefore, necessity of evaluation for the potential risk, such as allergenicity, for the NEPs produced in the GM plant body of the transgrafted plants remains to be further discussed.

## 5. Conclusion

Here, we showed that a cytosolic LUC protein can be translocated beyond the graft junction of a grafted plant, both from the rootstock to scion and vice versa. A previous analysis of phloem exudates showed that the majority of proteins ranged in size from 20−70 kDa, and the translocation of these mobile proteins is controlled by plasmodesmata at the pericycle-endodermis boundary^17^^)^. Since the size of the LUC protein is 60 kDa, the translocation of LUC protein is therefore predictable. Because the structure and permeability of plasmodesmata can be modified by both pathogens and the host plant^37^^)^, further study of NEP translocation is necessary to establish the safety of using transgrafting fruits. One important point is that translocation from the GM plant body is not a continuous event and is dependent on the characteristics of GM plant bodies. Given these factors, it is difficult to statistically assess degree of NEP translocation in the transgrafted plants. In addition, as mentioned in our previous study^20^^)^, small unfavorable metabolites are transferred from the GM plant body to non-GM plant body. Therefore, the potential risks of the transgene product and small molecules of concerned toxicity should be assessed when the transgene-free fruits obtained from the non-GM plant bodies of the transgrafted plants are commercialized.

## Supplementary materials

## References

[r1] 1.Habibi F, Liu T, Folta K, Sarkhosh A. Physiological, biochemical, and molecular aspects of grafting in fruit trees. Hortic Res. 2022; 9: uhac032. . 10.1093/hr/uhac032 35184166 PMC8976691

[r2] 2.Melnyk CW, Meyerowitz EM. Plant grafting. Curr Biol. 2015; 25(5): R183–R188. https://doi.org/10.1016/j.cub.2015.01.029. 25734263 10.1016/j.cub.2015.01.029

[r3] 3.Tworkoski T, Miller S. Rootstock effect on growth of apple scions with different growth habits. Sci Hortic (Amsterdam). 2007; 111(4): 335–343. . 10.1016/j.scienta.2006.10.034

[r4] 4.Jeynes-Cupper K, Catoni M. Long distance signalling and epigenetic changes in crop grafting. Front Plant Sci. 2023; 14: 1121704. . 10.3389/fpls.2023.1121704 37021313 PMC10067726

[r5] 5.Albacete A, Martínez-Andújar C, Martínez-Pérez A, Thompson AJ, Dodd IC, Pérez-Alfocea F. Unravelling rootstockxscion interactions to improve food security. J Exp Bot. 2015; 66(8): 2211–2226. . 10.1093/jxb/erv027 25754404 PMC4986720

[r6] 6.Lusser M, Parisi C, Plan D, Rodríguez-Cerezo E. Deployment of new biotechnologies in plant breeding. Nat Biotechnol. 2012; 30(3): 231–239. . 10.1038/nbt.2142 22398616 PMC7097357

[7] Zaidi SSA, Vanderschuren H, Qaim M, et al. New plant breeding technologies for food security. Science. 2019; 363(6434): 1390–1391. . 10.1126/science.aav6316 30923209

[r8] 8.Oshiro N, Kuniyoshi K, Nakamura A, Araki Y, Tamanaha K, Inafuku Y. A case of food poisoning due to ingestion of eggplant, Solanum melongena, grafted on Devil’s trumpet, Datura metel [In Japanese]. Shokuhin Eiseigaku Zasshi. 2008; 49(5): 376–379. . 10.3358/shokueishi.49.376 19029791

[9] Nakajima K, Hashimoto T. Two tropinone reductases, that catalyze opposite stereospecific reductions in tropane alkaloid biosynthesis, are localized in plant root with different cell-specific patterns. Plant Cell Physiol. 1999; 40(11): 1099–1107. . 10.1093/oxfordjournals.pcp.a029494 10635114

[10] De Storme N, Geelen D. Callose homeostasis at plasmodesmata: molecular regulators and developmental relevance. Front Plant Sci. 2014; 5: 138. . 10.3389/fpls.2014.00138 24795733 PMC4001042

[11] Melnyk CW, Schuster C, Leyser O, et al. A developmental framework for graft formation and vascular reconnection in *Arabidopsis thaliana*. Curr. Biol. 2015; 25(10): 1306–1318. https://doi.org/10.1016/j.cub.2015.03.032. 25891401 10.1016/j.cub.2015.03.032 PMC4798781

[12] Oparka KJ, Roberts AG, Boevink P, et al. Simple, but not branched, plasmodesmata allow the nonspecific trafficking of proteins in developing tobacco leaves. Cell. 1999; 97(6): 743–754. . 10.1016/S0092-8674(00)80786-2 10380926

[13] Corbesier L, Vincent C, Jang S, et al. FT protein movement contributes to long-distance signaling in floral induction of Arabidopsis. Science. 2007; 316(5827): 1030–1033. . 10.1126/science.1141752 17446353

[14] Navarro C, Abelenda JA, Cruz-Oró E, et al. Control of flowering and storage organ formation in potato by FLOWERING LOCUS T. Nature. 2011; 478(7367): 119–122. . 10.1038/nature10431 21947007

[15] Lifschitz E, Eviatar T, Rozman A, et al. The tomato *FT* ortholog triggers systemic signals that regulate growth and flowering and substitute for diverse environmental stimuli. Proc Natl Acad Sci USA. 2006; 103(16): 6398–6403. . 10.1073/pnas.0601620103 16606827 PMC1458889

[16] Yoo SC, Chen C, Rojas M, et al. Phloem long‐distance delivery of FLOWERING LOCUS T (FT) to the apex. Plant J. 2013; 75(3): 456–468. . 10.1111/tpj.12213 23607279

[17] Paultre DSG, Gustin MP, Molnar A, Oparka KJ. Lost in transit: long-distance trafficking and phloem unloading of protein signals in Arabidopsis homografts. Plant Cell. 2016; 28(9): 2016–2025. . 10.1105/tpc.16.00249 27600534 PMC5059797

[18] Kodama H, Miyahara T, Oguchi T, et al. Effect of transgenic rootstock grafting on the omics profiles in tomato. Food Safety. 2021; 9(2): 32–47. . 10.14252/foodsafetyfscj.D-20-00032 34249588 PMC8254850

[19] Miyahara T, Nishiuchi T, Fujikawa N, et al. Omics profiles of non-GM tubers from transgrafted potato with a GM scion. Food Safety. 2023; 11(1): 1–20. . 10.14252/foodsafetyfscj.D-22-00010 36970308 PMC10034357

[20] Ogawa T, Kato K, Asuka H, et al. Multi-omics analyses of non-GM tomato scion engrafted on GM rootstocks. Food Safety. 2023; 11(3): 41–53. . 10.14252/foodsafetyfscj.D-23-00005 37745161 PMC10514396

[r21] 21.Codex Alimentarius Commission (CAC). Joint FAO/WHO Food Standards Program. https://apps.who.int/iris/handle/10665/136259. Accessed on August 28, 2023.

[22] Goldschmidt EE. Plant grafting: new mechanisms, evolutionary implications. Front Plant Sci. 2014; 5: 727. . 10.3389/fpls.2014.00727 25566298 PMC4269114

[23] Nakamura S, Hondo K, Kawara T, et al. Conferring high‐temperature tolerance to nontransgenic tomato scions using graft transmission of RNA silencing of the fatty acid desaturase gene. Plant Biotechnol J. 2016; 14(2): 783–790. . 10.1111/pbi.12429 26132723 PMC11389092

[24] Rugini E, Silvestri C, Cristofori V, Brunori E, Biasi R. Ten years field trial observations of *ri*-TDNA cherry Colt rootstocks and their effect on grafted sweet cherry cv Lapins. Plant Cell Tissue Organ Cult. 2015; 123(3): 557–568. . 10.1007/s11240-015-0860-x

[r25] 25.Kodama H, Iwasa H, Hirai S, et al. Amplification of small interfering RNAs in transgenic plants. Agriculture Research and Technology. Bundgaard K, Isaksen L, eds. New York, USA: Nova Science Publishers; 2010: 379–395.

[26] Moore L, Warren G, Strobel G. Involvement of a plasmid in the hairy root disease of plants caused by *Agrobacterium rhizogenes*. Plasmid. 1979; 2(4): 617–626. . 10.1016/0147-619X(79)90059-3 231271

[27] White FF, Taylor BH, Huffman GA, Gordon MP, Nester EW. Molecular and genetic analysis of the transferred DNA regions of the root-inducing plasmid of *Agrobacterium rhizogenes*. J Bacteriol. 1985; 164(1): 33–44. . 10.1128/jb.164.1.33-44.1985 4044524 PMC214207

[28] Koizumi M, Shimotori Y, Saeki Y, Hirai S, Oka S, Kodama H. Effects of the 2b protein of *Cucumber mosaic virus* subgroup IB strain IA on different transgene-induced RNA silencing pathways. Plant Mol Biol Rep. 2017; 35(2): 265–272. . 10.1007/s11105-016-1020-0

[29] Giri A, Narasu ML. Transgenic hairy roots. recent trends and applications. Biotechnol Adv. 2000; 18(1): 1–22. . 10.1016/S0734-9750(99)00016-6 14538116

[30] Aragão MM, Alvarez MA, Caiafa L, Santos MO. Nicotiana hairy roots for recombinant protein expression, where to start? A systematic review. Mol Biol Rep. 2023; 50(5): 4587–4604. . 10.1007/s11033-023-08360-1 36917368

[31] McClain S, Herman RA, Islamovic E, et al. Allergy risk assessment for newly expressed proteins (NEPs) in genetically modified (GM) plants. J Reg Sci. 2021; 9(1): 67–75. . 10.21423/JRS-V09I1MCCLAIN

[32] Leclerc GM, Boockfor FR, Faught WJ, Frawley LS. Development of a destabilized firefly luciferase enzyme for measurement of gene expression. Biotechniques. 2000; 29(3): 590–591, 594–596, 598 passim. . 10.2144/00293rr02 10997273

[r33] 33.Noguchi T, Golden S. Bioluminescent and fluorescent reporters in circadian rhythm studies. *The Bioclock Studio*, Univ. California, San Diego, CA, USA, Mar. 2017. https://ccb.ucsd.edu/the-bioclock-studio/education-resources/reporter-review/ReporterReviewPDF.pdf. Accessed on August 28, 2023.

[34] Takeba G, Takimoto A. Translocation of the floral stimulus in *Pharbitis nil*. Bot Mag Tokyo. 1966; 79(942): 811–814. . 10.15281/jplantres1887.79.811

[35] Savage JA, Zwieniecki MA, Holbrook NM. Phloem transport velocity varies over time and among vascular bundles during early cucumber seedling development. Plant Physiol. 2013; 163(3): 1409–1418. . 10.1104/pp.113.225359 24072581 PMC3813660

[36] Moneret-Vautrin DA, Kanny G. Update on threshold doses of food allergens: implications for patients and the food industry. Curr Opin Allergy Clin Immunol. 2004; 4(3): 215–219. . 10.1097/00130832-200406000-00014 15126945

[37] Liu J, Zhang L, Yan D. Plasmodesmata-involved battle against pathogens and potential strategies for strengthening hosts. Front Plant Sci. 2021; 12: 644870. . 10.3389/fpls.2021.644870 34149749 PMC8210831

