# Supplementary material for: Discontinuous Translocation of a Luciferase Protein beyond Graft Junction in Tobacco
Source: Food Saf (Tokyo). 2024 Mar 22;12(1):1–16. doi: 10.14252/foodsafetyfscj.D-23-00010 (PMC10961615; doi:10.14252/foodsafetyfscj.D-23-00010)
Supplement: Supplementary file 1 [file foodsafetyfscj-12-1-s001.pdf]

Supplementary materials

Table S1. Summary of the number of raw reads, processed reads, and mapping rate of the transcriptomic data.

Sample name	Raw reads (count)	Processed reads (count)	Mapping rate* (%)
WT-LUC-1	77,827,994	77,221,978	87.55
WT-LUC-2	83,869,712	83,496,942	89.31

* Mapping rate was the alignment ratio to the *Nicotiana tabacum* cDNA sequences generated using genome assembly data (Ntab-TN90_AYMY-SS_NGS.mrna.annot.fasta).

doi: 10.14252/foodsafetyfscj.D-23-00010

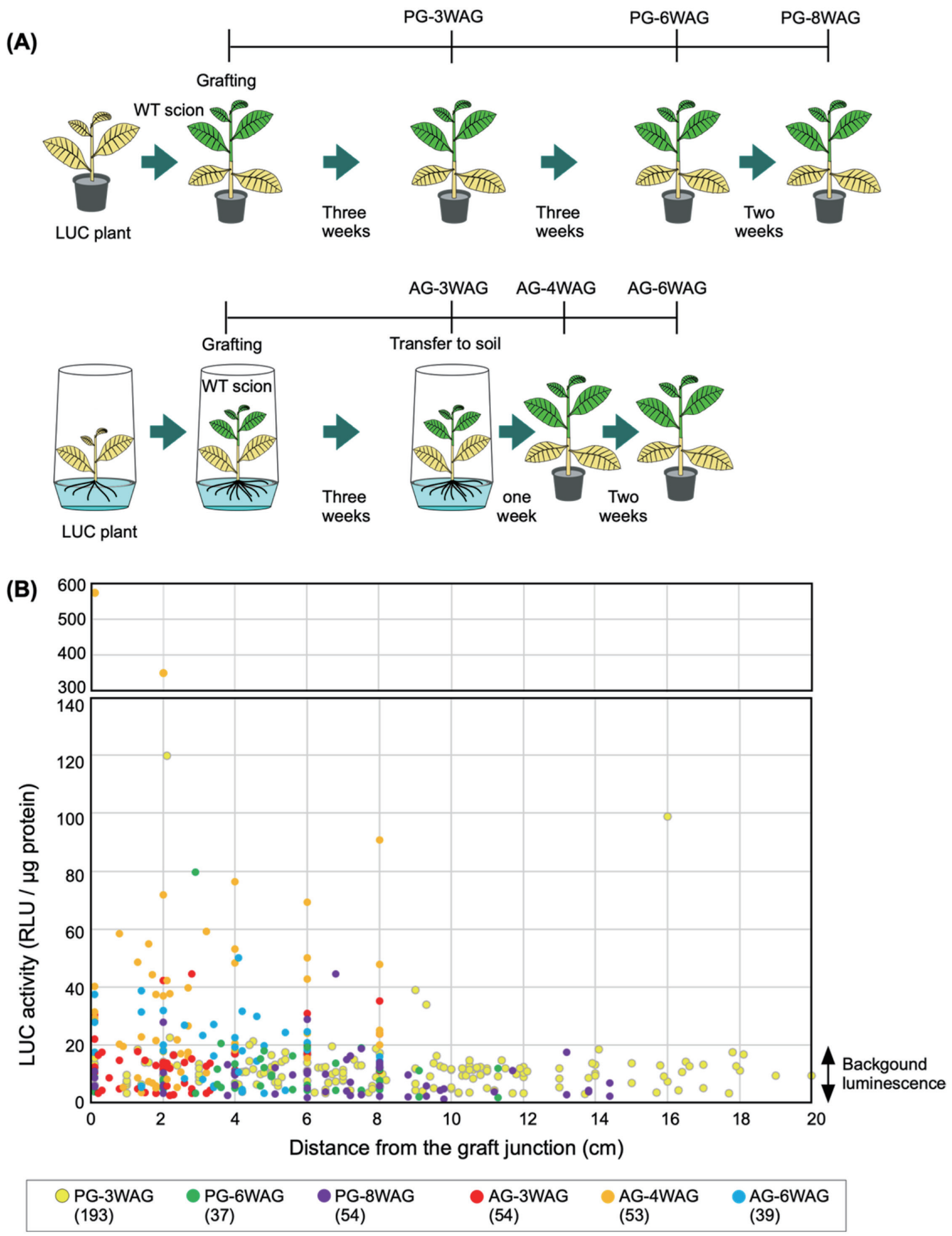

Fig. S1. Time-dependent movement of luciferase (LUC) protein from the rootstock. (A) A diagram showing the experimental transgrafting scheme. Two kinds of transgrafting protocol are shown. In the upper grafting method, LUC plants were grown on soil and WT plants were grafted onto them. Scion samples were then prepared 3, 6, and 8 WAG, and called PG-3WAG, PG-6WAG, and PG-8WAG, respectively. In the lower grafting method, LUC plants were aseptically grown in plant culture vessels. Next, aseptically grown WT scions were grafted onto them. These scion samples were prepared 3, 4, and 6 WAG, and were therefore named AG-3WAG, AG-4WAG, and AG-6WAG, respectively. (B) LUC activity measurements of scion samples prepared from WT/LUC transgrafted plants. The leaf, petiole, and stem tissues of the scion were sampled, and their corresponding LUC activities were plotted against the distance from the graft junction. The luminescence under 20 RLU / μg protein was accounted for as the background luminescence of tobacco tissue (see also **Figure 1** of the main text). Two scion samples from the AG-4WAG group showed extremely high LUC activities (shown in an extra panel). Data for the leaf and stem samples of PG-3WAG, PG-6WAG, and PG-8WAG are shown as **Figure 1** in the main text. The number of measurements used are shown in parentheses.

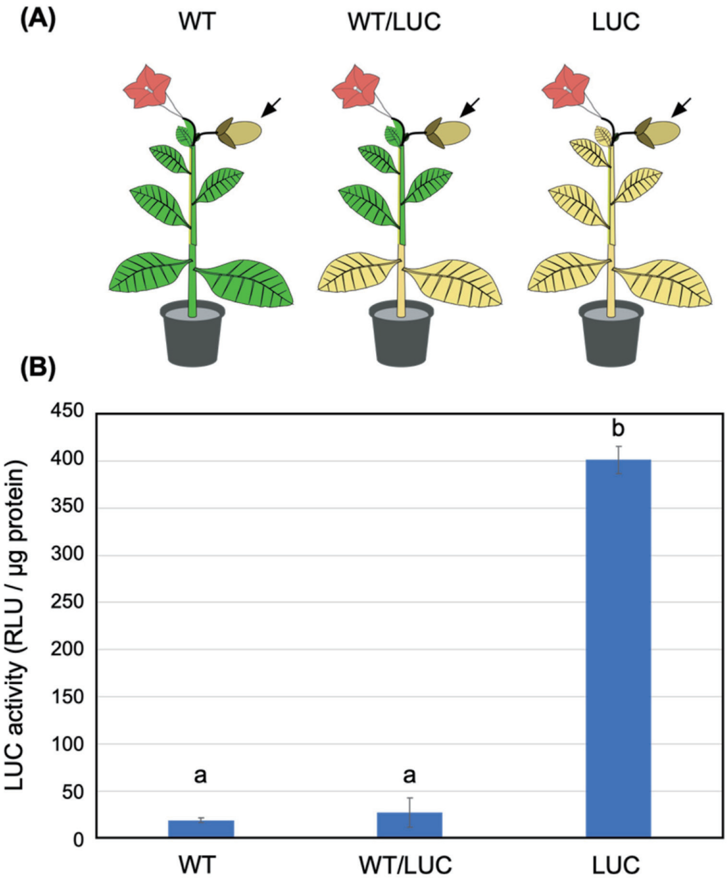

Fig. S2. LUC activity in seeds harvested from the WT scion of transgrafted plants with LUC rootstocks. (A) Diagram of seed preparation. WT (left) and LUC plants (right) were grown without grafting, and a WT/LUC plant was prepared by grafting a WT scion onto a LUC rootstock. (B) LUC activity of the seeds. Each crude protein extract was prepared by homogenization using 10 seeds per sample. All data are shown as a mean ± SD of three samples. The different letters indicate significant differences in LUC activity ($p < 0.05$).

doi: 10.14252/foodsafetyfscj.D-23-00010

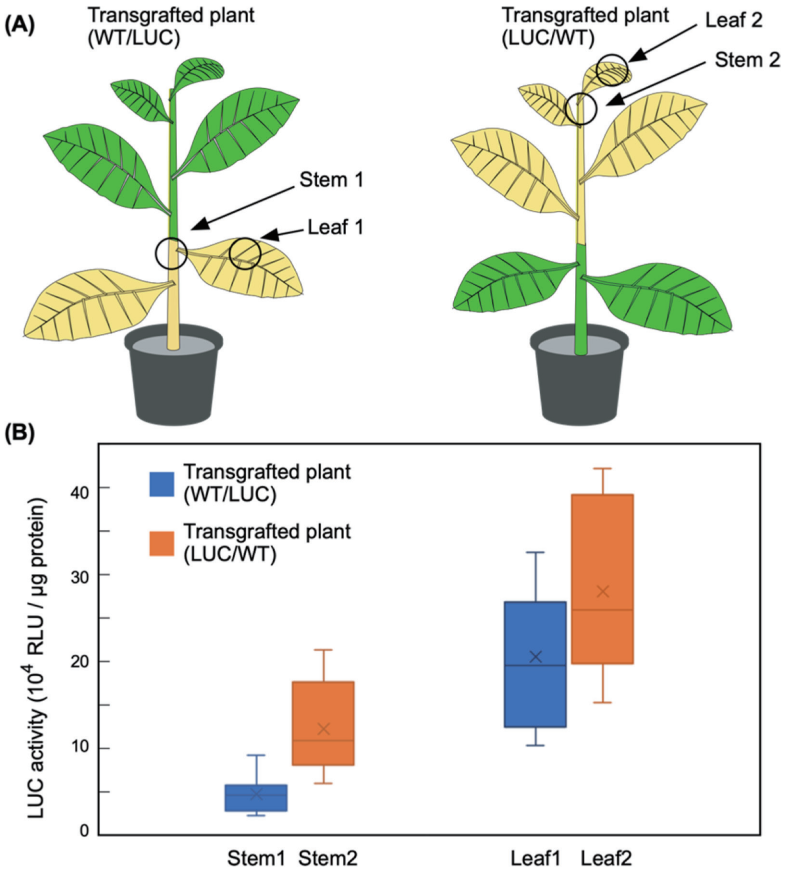

Fig. S3. Luciferase activity during transgrafting. (A) Tissues used for the determination of LUC activity. When LUC plants were used as the rootstock, we used the youngest tissue of the rootstock for the graft junction. Samples of "Stem1" were harvested 2 cm below the graft junction, and samples of "Leaf 1" were prepared from the leaf nearest to the graft junction. In contrast, when LUC plants were used as the scion, the youngest leaf and stem tissues were found near the shoot apical meristem (i.e., Stem 2 and Leaf 2). (B) LUC activities of prepared samples. These data indicate that the LUC scion produces more LUC protein compared to the LUC rootstock. The number of measurements for Stem 1 and 2 are 10 and 8, respectively. The number of measurements for Leaf 1 and Leaf 2 were also 10 and 8, respectively. In this box-and-whisker diagram, the box represents the first and third quartiles of the dataset. Moreover, the top and bottom ends of the whisker represent the maximum and minimum data points, respectively. The middle line represents the median, and "×" represents the mean.

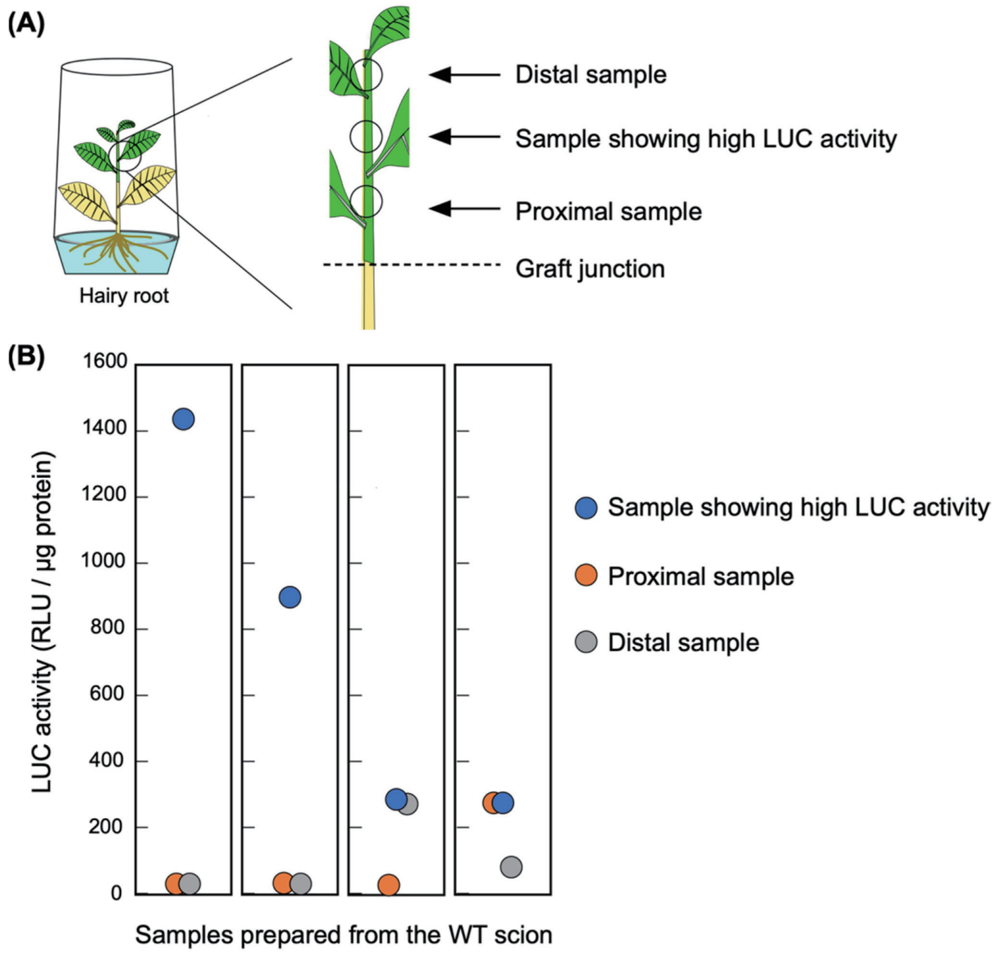

Fig. S4. Localized accumulation of luciferase protein in the scions of transgrafted plants. (A) Diagram showing the tissues used for the determination of LUC activity. (B) The LUC activities of stem samples prepared from the single scion of a WT/LUC transgrafted plant. LUC activity was determined using three successive samples. The sample showing the highest LUC activity was shown as a blue circle, and those of two flanking samples, (i.e., proximal and distal) were shown as orange and gray circles, respectively. Data shown was obtained from all four independent transgrafted plants.
